# Computational analysis of the roles of ER-Golgi network in the cell cycle

**DOI:** 10.1186/1752-0509-8-S4-S3

**Published:** 2014-12-08

**Authors:** Haijun Gong, Lu Feng

**Affiliations:** 1 Department of Mathematics and Computer Science, Saint Louis University, St. Louis, MO, 63103 USA

## Abstract

**Background:**

ER-Golgi network plays an important role in the processing, sorting and transport of proteins, and it's also a site for many signaling pathways that regulate the cell cycle. Accumulating evidence suggests that, the stressed ER and malfunction of Golgi apparatus are associated with the pathogenesis of cancer and Alzheimer's disease (AD). Our previous work discovered and verified that altering the expression levels of target SNARE and GEF could modulate the size of Golgi apparatus. Moreover, Golgi's structure and size undergo dramatic changes during the development of several diseases. It is of importance to investigate the roles of ER-Golgi network in the cell cycle progression and some diseases.

**Results:**

In this work, we first develop a computational model to study the ER stress-induced and Golgi-related apoptosis-survival signaling pathways. Then, we propose and apply both asynchronous and synchronous model checking methods, which extend our previous verification technique, to automatically and formally analyze the ER-Golgi-regulated signaling pathways in the cell cycle progression through verifying some computation tree temporal logic formulas.

**Conclusions:**

The proposed asynchronous and synchronous verification technique has advantages for large network analysis and verification over traditional simulation methods. Using the model checking method, we verified several Alzheimer's disease and cancer-related properties, and also identified important proteins (NFκB, ATF4, ASK1 and TRAF2) in the ER-Golgi network, which might be responsible for the pathogenesis of cancer and AD. Our studies indicate that targeting the ER stress-induced and Golgi-related pathways might serve as potent therapeutic targets for the treatment of cancer and Alzheimer's disease.

## Background

The pathogenesis of cancer and Alzheimer's disease (AD) is partially driven by the accumulation of genetic/epigenetic alterations and deregulation of important signaling pathways [1,2]. Alzheimer's disease is a common neurodegenerative disease in the elderly, which is characterized by the abnormal aggregation and deposition of misfolded proteins, and one hallmark of AD is the accumulation of beta-amyloid plaques. Understanding of the signaling mechanism will provide insights into the pathogenesis of AD and cancer. Though some targeted therapies could slow AD progression and tumor growth in some clinical studies, we still have not developed effective treatments for these two types of disease. Modern sequencing technology makes it easy to measure the gene expression data of cancer and Alzheimer's disease in a fast and precise way. The big challenge is how to identify and analyze the genetic signatures and important regulatory networks underlying the biological processes.

The endoplasmic reticulum (ER) and Golgi apparatus are two important organelles in the cell that play key roles in the assembling, folding, sorting and transport of newly synthesized secretory and transmembrane proteins in the final stages of biosynthesis. ER-Golgi network is also a site for many signaling pathways that regulate the cell cycle progression. Recent studies [1-6] indicate that, the ER stress-induced signaling pathways and malfunction of Golgi apparatus are associated with the pathogenesis of cancer and Alzheimer's disease. ER organelle's function can be disrupted by various intracellular and extracellular stimuli. The external stimuli and some genetic mutations can lead to abnormal accumulation of misfolded proteins on the ER and Golgi, inducing ER stress and Golgi malfunction [1,6]. The unfolded protein response (UPR) is a self-protective mechanism, which can promote cell survival in response to ER stress. And, the malfunction of UPR will also activate the apoptosis signaling pathway - a programmed cell death. Dysfunction of the UPR is associated with several diseases, including cancer and neurodegenerative disease (e.g., AD). Wlodkowic *et al*'s work [2] shows that, the secretory pathway regulated by the ER and Golgi apparatus can sense the external stress or stimulus, possibly leading to the activation of both survival and apoptosis signaling pathways if the stress-signaling threshold is exceeded. So, targeting some ER stress-induced and Golgi-related apoptosis-survival signaling pathways and proteins could be novel therapeutic targets for cancer and AD treatment.

Our previous work [7,8] based on discrete stochastic simulation methods found that, changing the expression level of GEF and tSNARE proteins in the ER-Golgi network could modulate the size of Golgi apparatus. Moreover, Golgi's function and size undergo dramatic changes during the development of several diseases [2]. Our objective is to study the roles of ER-Golgi network in the cell cycle progression, investigate the molecular mechanisms of the ER stress-induced apoptosis-survival pathways in the pathogenesis of cancer and AD. Different computational models (e.g., discrete value, ordinary (stochastic) differential equations, and Gillespie's stochastic simulation) will be helpful to study the roles of ER-Golgi network in the cell cycle progression and some diseases. Since the signaling network of cancer and AD is complex, and many parameters of biochemical reactions are not known, traditional simulation methods can not correctly and efficiently study such a complex model. Our previous work proposed and applied Statistical Model Checking [9,10] and synchronous Symbolic Model Checking [11-14] techniques to study the signaling pathways in the cancer cell. In these work [11-14] we had assumed all the reactions occur synchronously, i.e., the state of each protein (node) is updated at the same time. Though several properties predicted by the synchronous model checker are consistent with existing experiments, this assumption has received critics from some reviewers and other researchers. This is due to the fact that biochemical reactions in the cell normally evolve at different rates, that is, the state of each protein (node) can not be updated at the same time. So, synchronous models might not be able to correctly describe the temporal behaviors of some cellular components.

In this work, we will extend our previous synchronous verification technique and propose an asynchronous model checking method to formally analyze the ER-Golgi-regulated signaling pathways. With the help of asynchronous and synchronous model checker, we construct and check some computation tree temporal logic formulas related to the cancer and Alzheimer's disease and study the roles of ER-Golgi network in the cell cycle progression. A comprehensive understanding of the signaling pathways regulated by ER and Golgi apparatus will help discover the mechanisms underlying cancer and AD, and develop some possible targeted therapies through regulating the function of ER-Golgi network.

## Methods

One of the most important signaling pathways regulated by the ER-Golgi network is the unfolded protein response (UPR) pathway. UPR is initiated by three ER membrane-associated transmembrane-proteins: ATF6 (activating transcription factor-6), PERK (Protein kinase RNA-like ER kinase) and IRE1 (inositol-requiring transmembrane kinase/endoribonuclease-1), which can sense the accumulation of misfolded proteins on the ER. Dysfunction of the UPR pathway can disrupt ER homeostasis and influence the pathogenesis of cancer, diabetes and neurodegenerative disease (e.g., AD), etc.

We will first summarize the ER stress-induced and Golgi-related signaling pathways. Our objective is to investigate the roles of ER-Golgi network and identify key signaling components that regulate the cell cycle progression, especially how it is associated with the pathogenesis of cancer and Alzheimer's disease.

### Signaling pathways regulated by the ER-Golgi network

**Figure 1:**
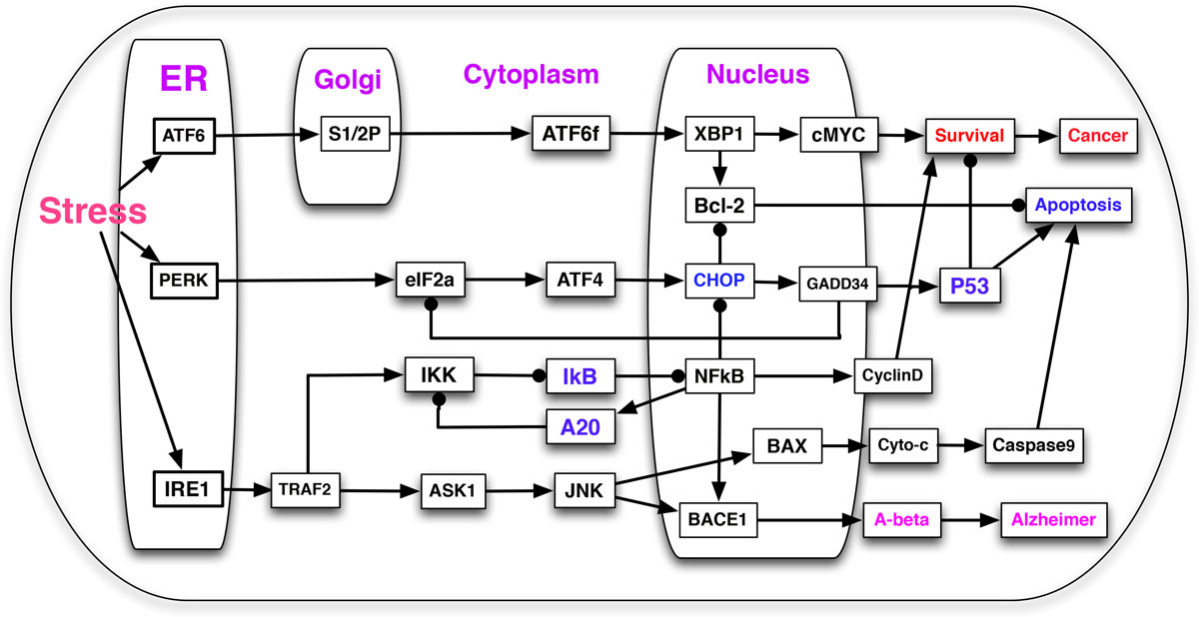
**ER stress-induced and Golgi-related signaling pathways in the cell cycle**. The proposed network includes the input nodes (ATF6, PERK, IRE1) on the ER and S1P/S2P on the Golgi due to ER stress, and 3 output nodes (cell's fate: Cancer, Apoptosis, Alzheimer). The symbol → denotes activation, while  ⊣denotes inhibition, except ATF6 → S1/2P → ATF6f which represents that, ATF6 enters the Golgi and it is processed by S1/2P, leading to the final product ATF6f.

We performed an extensive literature search to construct a model of signaling pathways initiated by ATF6, PERK and IRE1 sensors, which is depicted in Figure 1. The symbol → denotes activation, while  ⊣ denotes inhibition, except "ATF6 → S1/2P → ATF6f" on the Figure 1 which will be discussed in detail later.

ATF6 is an ubiquitously expressed ER transmembrane protein. In response to ER stress or abnormal accumulation of misfolded proteins on the ER, ATF6 will be transported to the Golgi apparatus through its interaction with the coat protein II (COPII) complex [1], which is regulated by the GEF and tSNARE mechanism validated in our previous Golgi research [7,8]. The site 1 protease (S1P) and S2P in the Golgi apparatus will process ATF6 and release its N-terminal cytosolic domain fragment (ATF6f) to the cytoplasm. After translocating to the nucleus, the synthesized ATF6f will rapidly activate several downstream genes [1,15], including the X-box-binding protein 1 (XBP1), which upregulates the expressoin of cMYC and Bcl-2, leading to the cell survival and inhibition of apoptosis. This pathway is summarized as: ATF6 + S1/2P → ATF6f →XBP1→{cMYC, Bcl-2}→Survival.

PERK is another ER transmembrane sensor which can detect the abnormal accumulation of misfolded proteins at its N-terminal domain. The activated PERK will phosphorylate the α-subunit of eIF2 (eukaryotic translation initiation factor-2), which can induce the translation of ATF4 (activating transcription factor-4). ATF4 then translocates to the nuclues to activate the transcription of some apoptosis-related genes [1,15]. Particularly, ATF4 can upregulate the expression of CHOP, which in turn activates the transcription of GADD34, leading to the activation of P53-dependent apoptosis signaling pathway [16]. eIF2 defects are lethal in many diseases due to its essential roles in the protein synthesis, and eIF2 has been found to be down-regulated (inactive) in patients suffering from cancer and neurodegenerative diseases [17], including Alzheimer's disease. The PERK pathway is summarized as: PERK → eIF2α → ATF4 → CHOP → GADD34 → P53 → Apoptosis.

IRE1 is also an ER transmembrane protein with kinase and endoribonuclease (RNase) activity in its C-terminal cytosolic domain [1,15]. Under ER stress, IRE1 can activate three signaling pathways involved in the apoptosis, survival and Amyloid-β production. (1) IRE1 → TRAF2 → ASK1 → JNK → BACE1 → Aβ → Alzheimer: IRE1 interacts with TRAF2 (tumor necrosis factor receptor associated factor-2) to recruit and activate ASK1 (Apoptosis signal-regulating kinase 1), leading to the activation of JNK pathway [1,3]. Studies in Alzheimer's disease found, the JNK pathway could promote the production of Amyloid-β (Aβ) through activating the expression of BACE1 (beta-secretase). The aggregation of Aβ, which is the main component of amyloid plaques found in the brains of Alzheimer patients, is one of the major hallmarks in Alzheimer's disease. (2) TRAF2 → IKK  ⊣ IκB  ⊣ NFκB → {CyclinD, BACE1, A20}: TRAF2 phosphorylation can also activate the NFκB pathway. In the resting cell, NFκB is inhibited by IκB, a tumor suppressor, by forming a complex and stay in the cytoplasm. Once IKK (IκB kinase) is activated by TRAF2, the complex will dissemble and NFκB will translocate into the nucleus to promote the transcription of oncoproteins (Cyclin D, BACE1) and tumor suppressor A20 [18,19], which can inhibit IKK's activity. (3) IRE1-JNK pathway could also induce the cytochrome c (Cyto-c) release and caspase-dependent apoptosis pathway: JNK → BAX → Cytoc → Caspase9 → Apoptosis.

### Simulation model

Computational analysis of the ER stress-induced survival and apoptosis signaling pathways could help identify therapeutic targets and develop drugs for the treatment of cancer and Alzheimer's disease. Figure 1 describes the crosstalk of different signaling pathways regulated by the ER-Golgi network in the cell cycle due to ER stress. The proposed network is composed of *m *= 30 nodes, including input nodes (ATF6, PERK and IRE1 are directly activated by the ER stress) in the ER and S1/2P in the Golgi apparatus. In this work, we will use Boolean variables of "Cancer", "Apoptosis" and "Alzheimer" to represent the fates of the cell in various conditions (that is, three possible outputs). If each node in the model takes *n *possible discrete values, the model can have up to $n^{m}$ possible configurations/states. If the number of nodes *m *is very large, it will lead to a state explosion problem (e.g., if the model has *m *= 30 nodes, *n *= 2, $2^{30}$≈ 1 billion; *n *= 3, $3^{30}$ ≈ 20 trillion!). Given a large network, one of the challenges for the systems biologists is how to simulate and verify it in a correct and effective way. Due to the state explosion problem and many unknown parameters, we have emphasized in our previous work [14] that, traditional simulation methods, including BooleaNet [20], ordinary (stochastic) differential equation [7] and Gillespie's stochastic simulation methods [9,10], can not effectively simulate large networks.

Our aim in this work is to qualitatively analyze the ER-Golgi-regulated signaling pathways and identify the genetic signatures and signal transduction sequence that will drive the cell to some diseases [11,14] (apoptosis, cancer or Alzheimer's disease). In [14], we developed a discrete value model to describe the expression levels of regulatory components on the signaling pathways in the tumor microenvironment. In this work, we will adopt this method and apply it to describe the ER-Golgi network. We assume each node can take *n *possible values (states or protein's expression levels) {*0, 1, 2, ..., n-1*}, and the state of each node from time *t *to *t + 1 *is updated by a transfer function decided by its parent nodes which are classified as activators and inhibitors [11,12,14]. The state transfer function for a given node $X_{n}$ at time *t + 1 *regulated by both activators $A_{i}$ and inhibitors $I_{j}$, is written as

$$X_{n}\left(t+1\right)=\left\{\begin{array}{c} n-1\;\;\;if\;\underset{i}{\sum }A_{i}\left(t\right)-\underset{j}{\sum }I_{j}\left(t\right)\geq n-1\\ \ldots \ldots \\ 2\;\;\;if\;\underset{i}{\sum }A_{i}\left(t\right)-\underset{j}{\sum }I_{j}\left(t\right)=2\\ 1\;\;\;if\;\underset{i}{\sum }A_{i}\left(t\right)-\underset{j}{\sum }I_{j}\left(t\right)=1\\ 0\;\;\;if\;\underset{i}{\sum }A_{i}\left(t\right)-\underset{j}{\sum }I_{j}\left(t\right)\leq 0, \end{array}\right)$$

where, $A_{i}\left(t\right)$ and $I_{j}\left(t\right)$ represent the states of activators and inhibitors at time *t *respectively. Similar to [14], a three-value model is implemented to analyze the temporal and dynamic behaviors of the cell in response to ER stress and Golgi dysfunction. If we assume the states of all proteins are updated synchronously by the transfer function, then, this model will have at least 20 trillion possible configurations/states in the state transition diagram.

In this work, one of the novelties is, we are building an asynchronous model of ER-Golgi network, that is, all the reactions on the signaling pathway occur at different rate and nondeterministically. So, the number of states and processes to be checked in the asynchronous model will be considerably increased compared with the synchronous model, leading to a serious state explosion problem, which will be discussed in the next section. Now, the big challenge facing us is, how to search and verify a network with an astronomical number of states? To solve this problem, we introduce the synchronous and asynchronous Symbolic Model Checking in the next Section.

### Model checking and temporal logics

Model Checking, a formal verification technique [21], can automatically verify or falsify that a model *M *satisfies a desired property expressed as a temporal logic formula  ψ, denoted by M⊨ψ. Model Checking has been successfully applied to verify hardware and software systems. Recently, it was applied to study the complex biological network in our work [9-14]. We have introduced the semantics in our previous work [9,10,14], for completeness sake, we will briefly discuss the fundamentals of Model Checking in this work again.

A Kripke structure, which is a tuple $M=\left(S,S_{0},R,L\right)$, represents the finite state transition system in model checking, where, $S_{0}$ denotes a set of initial states,  R denotes a transition relation between states, and  L denotes a function that labels each state *s *with a set of atomic propositions true in *s*. Given a Kripke structure (a model) *M *and a temporal logic formula  ψ, Model Checking algorithms automatically and exhaustively search the state space to find all states that satisfy  ψ. If the property  ψ is not satisfied, a counterexample that falsifies  ψ will be given by the model checker; else, we'll say *M *satisfies  ψ.

Next, we will translate the experimental phenomenon or some desired property into a temporal logic formula. Computation Tree Logic (CTL) formulas describe the properties of computation trees, whose root corresponds to the initial state $S_{0}$. The branches and leaves represent all possible sequences of state transitions (paths) from the root [21]. A CTL formula is constructed from atomic propositions, Boolean logic connectives, temporal operators (**X**, **F**, **G**, **U**) describing the temporal properties on a path, and two path quantifiers (**A**, **E**). The following operators have been frequently used in our work to construct a CTL formula: **AG**p - p is **G**lobally true on **A**ll paths; **EG**p - there **E**xists a path where p is globally true; **AF**p - p holds at some state in the **F**uture on all paths; **EF**p - there exists a path where p holds at some state eventually; **AX**p - p holds in the ne**X**t state on all the paths; **EX**p - there exists a path where p holds in the next state. The temporal and dynamic behaviors of the signaling pathway will be expressed as CTL formulas in this work. For example, the formula **AG**(ASK1 = 2 → **AF**(Aβ ≥ 1 & Alzheimer = True)) means, whenever the ASK1 is activated or overexpressed (taking a value 2), it will always promote the synthesis of Aβ and induce the pathogenesis of Alzheimer's disease eventually. The syntax and semantics of CTL logic have been defined in [21], the readers could refer to our recent work [14] for details.

### Synchronous symbolic model checking

During formal verification, model checker can automatically and exhaustively search the state transition system *M *to verify or falsify the specified temporal logic formula  ψ. In our recent work [14], we introduced and applied a synchronous Symbolic Model Checker to study the signaling pathways in the tumor microenvironment described by a discrete value model. Symbolic Model Verifier (SMV) [22], which is based on a data structure called ordered binary decision diagram [23], can automatically verify CTL formulas and output "True" (if the property is satisfied) or "False" with a counterexample (if the property is not satisfied). Compared with traditional model checker, SMV represented the transition relation implicitly using a Boolean function in order to overcome the state-space explosion problem. The detailed synchronous Symbolic Model Checking algorithm can be found in [14,22].

**Figure 2:**
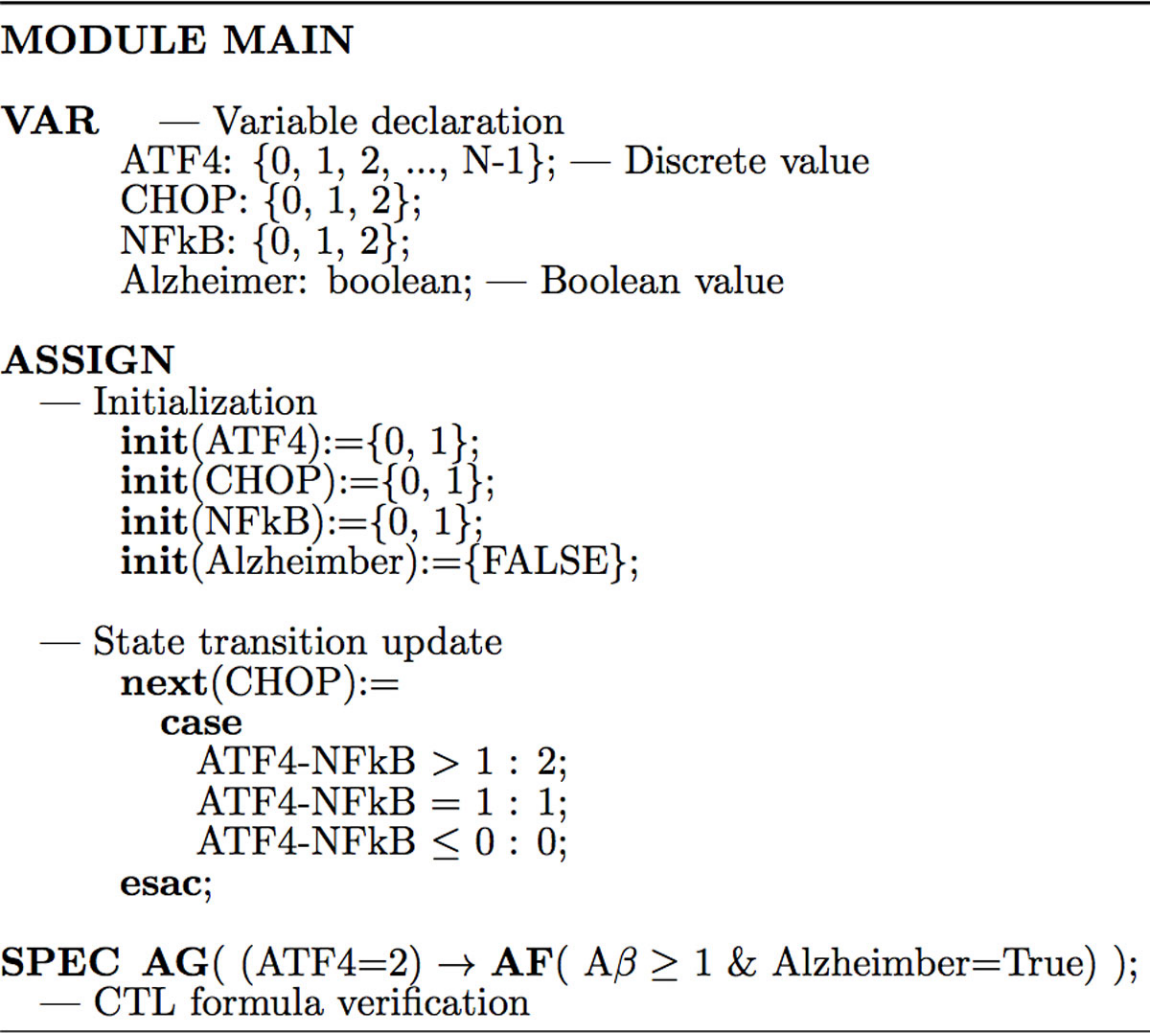
**Synchronous SMV code for the ER-Golgi network model checking**. The synchronous SMV program consists of one main module. All the variable are declared with the keyword VAR. The initial values (init) and state transition update (next) are defined with the keyword "ASSIGN". The CTL formula is encoded with the keyword "SPEC".

Similar to [11,12,14], Figure 2 demonstrates the procedure to write the SMV code of synchronous model checker and verify CTL formulas related to the ER stress-induced and Golgi-related signaling pathways. In the synchronous SMV, the program consists of only one module, that is, "**MODULE MAIN**", and all the variables are declared with the keyword **VAR**. The initial values (**init**) and state transition update (**next**) will be defined under the keyword **ASSIGN**. For example, "init(ASK1) = {0,1}" means, ASK1 can take a value of either 0 or 1 (with a probability) initially. The CTL formula is encoded and to be verified with the keyword "**SPEC**". For example, the statement "**SPEC AG**(ASK1 = 2 → **AF**(Alzheimer=True))" means, overexpressed ASK1 will eventually induce the pathogenesis of Alzheimer disease on all paths. The synchronous SMV model checker will automatically implement the Symbolic Model Checking algorithm [14]. The complete synchronous SMV code is available at [24].

In the synchronous model checking, we assume that all the events occur synchronously, i.e., the state of each node is updated at the same time. However, biochemical reactions in the cell are stochastic processes and they normally occur at different rates (speeds), that is, the state of each node (protein) should be updated asynchronously. In the next section, we will discuss how to use an asynchronous SMV model checker to study the signaling pathways regulated by the ER-Golgi network.

### Asynchronous symbolic model checking

In the asynchronous Symbolic Model Checking, the SMV program can consist of several modules with or without parameters, and each module corresponds to one asynchronous process (which contains all the reactions that a specific regulatory component is involved in). But, similar to the synchronous SMV, every asynchronous SMV program must have one root module, called "**MODULE MAIN**" without any parameters, which forms the starting point to build a finite-state model [22].

**Figure 3:**
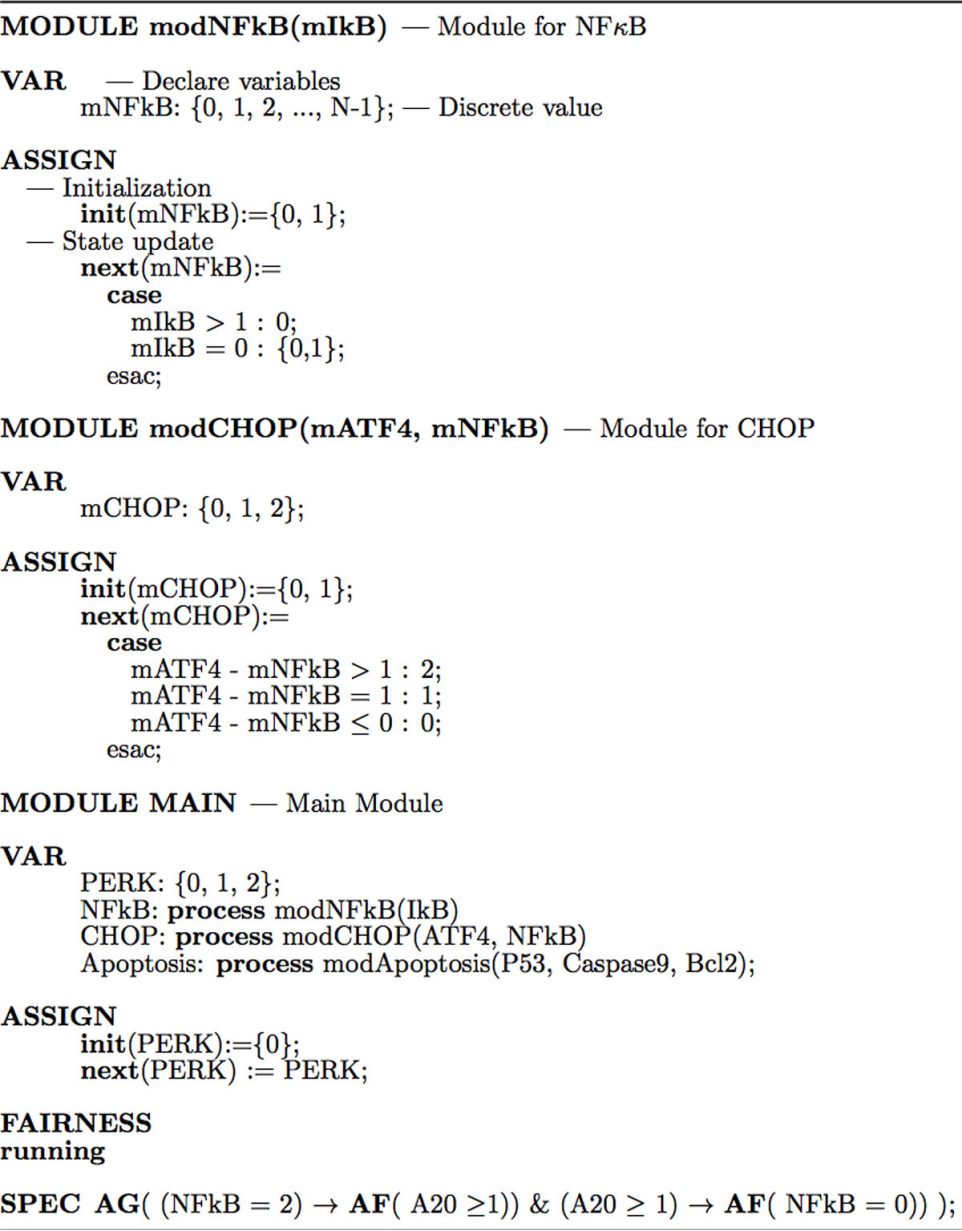
**Asynchronous SMV code for the ER-Golgi network model checking**. The asynchronous SMV program consists of more than one module with or without parameters besides the main module. Each asynchronous process/event for a declared variable should be instantiated using the keyword "process". The variable declaration, initialization and update in each module still use the keywords "VAR", "init" and "next" respectively.

Figure 3 illustrates the procedure to develop an asynchronous SMV program to study the signaling pathways regulated by the ER-Golgi network and verify CTL formulas. Each module in the program is an encapsulated description, and the variables (nodes) are passed into modules by reference. For example, "**MODULE **modCHOP(mATF4, mNFkB)" defines modCHOP as a module for the protein CHOP with 2 parameters "mATF4" and "mNFkB", which means the protein CHOP is regulated by both ATF4 and NFκB (two parent nodes). Since each variable is a state machine, so the variable declaration, initialization and update in each module still use the keywords **VAR**, **init **and **next **respectively. For example, the internal variable mCHOP can take discrete values of {0, 1, 2} in the module "modCHOP".

In the "main" module, each asynchronous process/event for a declared variable should be instantiated using the keyword "**process**", e.g., in Figure 3, "CHOP: **process **modCHOP(ATF4, NFkB)". SMV program will execute these processes asynchronously, that is, at each step, only one process will be non-deterministically chosen and executed from all the modules instantiated with the keyword "process". Then, all the assignment statements (with a keyword "**ASSIGN**") declared in that process are executed in parallel [22]. If some variable is not assigned by the "process", then its value remains unchanged, e.g., in Figure 3, the input node "PERK: {0,1,2}" will not be updated asynchronously. Because the asynchronous model checker non-deterministically choose and execute a process, there is a considerable increase in the complexity of the algorithm and number of transitions/processes to be checked compared with synchronous model checking. SMV could check up to $10^{100}$ possible states of synchronous and asynchronous models in several minutes which is a big advantage in the large network validation and analysis.

Since the asynchronous symbolic model checker does not force the system to eventually choose a given process (event) to execute, we have to use a fairness constraint by adding the following declaration: "**FAIRNESS running**", which can force the checker to execute a given process (event) infinitely often [22]. The variable "running" is 1 if and only if that process is currently executed during the model checking procedure. The full asynchronous SMV code is available on [24].

## Results and discussion

In this section, we will apply both synchronous and asynchronous model checkers to automatically analyze the signaling pathways illustrated in Figure 1. As to the regulatory components' initial values, we adopt the convention from our previous work [14], the internal nodes can be either 0 or 1 with different probabilities (the model checker chooses an initial value of either 0 or 1 nondeterministically), and the output nodes "Cancer", "Apoptosis" and "Alzheimer" are set to be False (OFF or 0). Some CTL formulas were abstracted from the *in vitro *or *in vivo *experiments in the cancer and Alzheimer's disease literatures, which will be used to formally verify the proposed model. We classify the temporal logic formulas into three categories that are related to Alzheimer's disease, cancer and oscillation behaviors. We aim to predict the cell's fate (Apoptosis, Alzheimer's disease and Cancer) in response to ER stress and Golgi dysfunction, identify genetic signatures that are associated with the pathogenesis of some diseases, and qualitatively analyze the dynamic behaviors (oscillation) of some regulatory components. Moreover, this work also predicts new properties which could be tested in the future cancer and Alzheimer's disease studies.

### Temporal logic formulas in Alzheimer's disease

First, we check some CTL formulas related to the cell's fate if the ER membrane-associated protein PERK or IRE1 is activated or overexpressed after sensoring the ER stress. That is, given a predefined initial condition (e.g., PERK = 2, IRE1 = 2), will the cell finally reach the state of Apoptosis or Alzheimer's disease? This type of CTL formula is also called reachability.

**Property 1: **IRE1 = 2 → **AF**(Aβ ≥ 1 & Alzheimer = True)

**Property 2: **PERK = 2 → **AF**(Aβ ≥ 1 & Alzheimer = True)

Properties 1-2 are checking, if initially the protein IRE1 or PERK is overexpressed due to ER stress, the cell will finally reach a state "Aβ ≥ 1 & Alzheimer = True" on all paths. That is, the production of Amyloid-β (Aβ) will eventually be induced, leading to the pathogenesis of Alzheimer's disease. These two formulas are verified to be true by both synchronous and asynchronous model checkers. These two properties are consistent with previous experimental observations [25,26], that is, ER stress promotes the production of amyloid-beta peptides, a main composition of amyloid plaques, which is a major hallmark of Alzheimer's disease.

**Property 3: **IRE1(PERK) = *j *→ **AF**(Caspase9 ≥ 1 & Apoptosis = True)

**Property 4: **IRE1(PERK) = 2 → **AF**(Aβ ≥ 1 & Alzheimer = True & Caspase9 ≥ 1 & Apoptosis = True)

Property 3 tests, if initially the protein IRE1 or PERK takes a value "*j*", could the cell, for all paths, finally reach the state "Caspase9 ≥ 1 & Apoptosis = True"? Our work shows, if *j *= 1 (activated), this property is false, that is, the caspase-dependent apoptosis can not always occur. But, if *j *= 2 (overexpressed, or prolonged ER stress), both synchronous and asynchronous SMV model checkers verified the property 3, that is, the cell will finally reach "apoptosis" on all paths. Both model checkers also verified the property 4 that, the prolonged ER stress can not only promote the pathogenesis of Alzheimer's diseases, but also induce the "Apoptosis".

Another interest in the studies of Alzheimer's disease, as a systems biologist, is to identify genetic signatures or regulatory components which can accelerate or slow the progression of AD with or without ER stress. We identified the following important players in the pathogenesis of AD.

**Property 5: AG**{(TRAF2 = 2) → **AF**(Aβ ≥ 1 & Alzheimer = True)}

**Property 6: AG**{(NFκB = 2) → **AF**(Aβ ≥ 1 & Alzheimer = True)}

**Property 7: AG**{(ASK1 = 2) → **AF **(Aβ ≥ 1 & Alzheimer = True)}

Properties 5-7 show that, if the internal node TRAF2 or ASK1 or NFκB is mutated or overexpressed (taking a value of 2), its downstream signaling components will be continuously activated on all paths in the future, finally promoting the synthesis of Aβ and pathogenesis of AD, even if there is no ER stress or external stimulus. These CTL formulas were verified to be true by synchronous SMV, meaning that these proteins and corresponding pathways might be possible therapeutic targets for the Alzheimer's disease. Our results are consistent with Culpan *et al*'s experiment [27], which revealed that TRAF2's expression levels increase in the brain of Alzheimer's disease. Previous studies also found that the signaling pathway regulated by NFκB [28] was involved in the pathogenesis of neurodegenerative disorders (e.g., AD).

**Property 5': AG**{(TRAF2 = 2) → **EF**(Aβ ≥ 1 & Alzheimer = True)}

**Property 6': AG **{(NFκB = 2) → **EF **(Aβ ≥ 1 & Alzheimer = True)}

**Property 7': AG **{(ASK1 = 2) → **EF **(Aβ ≥ 1 & Alzheimer = True)}

However, the properties 5-7 were falsified in the asynchronous model checking, which is not a surprise since asynchronous SMV searches all the possible sequences of reactions, including those sequences that never occur in the real world. But the properties 5'-7' were verified to be true by the asynchronous SMV, it means, there exists a path in the asynchronous model that the mutated TRAF2 or NFκB or ASK1 will eventually promote the pathogenesis of Alzheimer's disease.

### Temporal logic formulas in cancer

Similar to the studies of Alzheimer's disease, next, we proposed some reachability CTL formulas to study the precancerous or cancerous cell. If the initial value of ATF6 is set to be active, will the cell reach the state of "Cancer = True & Apoptosis = False"?

**Property 8: **ATF6 ≥ 1 → **AF**(cMYC ≥ 1 & Cancer = True & Apoptosis = False)

**Property 9: **(ATF6 ≥ 1 & S1/2P ≥ 1) → **AF**(cMYC ≥ 1 & Cancer = True & Apoptosis = False) **Property 9': **(ATF6 ≥ 1 & S1/2P ≥ 1) → **EF**(cMYC ≥ 1 & Cancer = True & Apoptosis = False)

In the property 8, we first check, under ER stress, could the activated ATF6 protein alone induce the cancer cell survival and inhibit apoptosis on all paths? This formula is falsified by both synchronous and asynchronous model checkers. This is due to the fact that, the ER transmembrane protein ATF6 must be pre-processed by S1P/S2P first in the Golgi apparatus to become an active form ATF6f (both proteins have contributions!) before it moves to nucleus to activate other genes' transcription and induce cell survival. So the active ATF6 alone can not promote the cell cycle progression. While, the property 9 shows that, the activation of both ATF6 and S1/2P will activate the onprotein cMYC, promote cancer cell survival and inhibit apoptosis. This property is verified by synchronous model checker but falsified by the asynchronous SMV. However, the property 9', which is weaker than property 9, is true in both model checkers.

**Property 10: **IRE1 = 2 → **AF**(CyclinD = 2 & Cancer = True & P53 = 2 & Apoptosis = True)

In the property 4, we verified that overexpressed IRE1 could induce caspase-dependent apoptosis in the AD cell. Property 10 claims, in a precancerous cell, overexpressed IRE1 can activate both survival and apoptosis pathways. This statement seems controversial. However it is verified by both model checkers, because the ER stress could activate both pro-survival and apoptosis mechanisms if the stress-signaling threshold is exceeded according to recent cancer studies [2]. Next, we will identify important regulatory components, including the oncoproteins and tumor suppressors, which can promote or inhibit the tumorigenesis. The following formulas were checked in both synchronous and asynchronous models.

**Property 11: AG**{(IKK = 2) → **AF**(P53 = 0 & Cancer = True)}

**Property 11': AG**{(IKK = 2) → **EF**(P53 = 0 & Cancer = True)}

**Property 12: AG**{(NFκB = 2) → **AF**(P53 = 0 & Cancer = True)}

**Property 12': AG**{(NFκB = 2) → **EF**(P53 = 0 & Cancer = True)}

**Property 13: AG**{(ATF4 = 2) → **AF**(P53 ≥ 1 & Apoptosis = True & Cancer = False)}

**Property 13': AG**{(ATF4 = 2) → **EF**(P53 ≥ 1 & Apoptosis = True & Cancer = False)}

Properties 11-12 (11'-12') identified two oncoproteins IKK and NFκB whose continuous activation or mutation/overexpression could eventually inhibit the expression of P53, an important tumor suppressor, and induce the cancer cell survival. Property 13 (13') identified one possible tumor suppressor, ATF4, which regulates MYC-mediated cell death [27]. These properties suggest novel avenues to inhibit tumorigenesis and promote apoptosis through inhibiting IKK-NFκB pathway, e.g., using the IKK inhibitor (Manumycin A), which has been confirmed in our previous work [10,11,14]. All these formulas were verified by the synchronous SMV model checkers, but only the properties 11'-13' were verified to be true by asynchronous checker. Property 13' means that, in the ATF4-treated (activated) cancer cells, there EXISTS a path such that the tumor suppressor P53 and apoptosis mechanism will be activated eventually. This is consistent with current experimental studies that some single-gene targeted therapies could inhibit tumor growth. The falsified property 13 by asynchronous model checking means, targeting ATF4-pathway alone in the cancer cell can NOT, for ALL paths, guarantee to induce apoptosis eventually. These properties, similar to the studies in [14], again, show the significant roles of the signaling-crosstalk among different pathways in the tumorigenesis.

From the above properties, we found NFκB is an important player in the pathogenesis of both cancer and Alzheimer's disease. Then, we checked the following formula which was verified by the synchronous SMV model checker only. Property 14 explains why NFκB has been taken as one potent therapeutic target in the cancer and AD treatment.

**Property 14: AG**{(NFκB = 2) → **AF**(P53 = 0 & Cancer = True & Aβ ≥ 1 & Alzheimer = True)}

### Oscillations of regulatory components

Experimental studies of some specific signaling pathways observed, the external stimulus could induce signal oscillations, which have attracted the attention of computational biologists [9,14]. Transcription factor NFκB regulates the transcription of several genes that are involved in the cancer, apoptosis, inflammation, and Alzheimer's disease that have been discussed in the last two sections. In the resting cell, IκB binds to NFκB to form a complex in the cytoplasm. In response to external stimulus, IκB kinase (IKK) will promote the degradation of IκB, leading to the translocation of NFκB to the nucleus. The activation of NFκB could induce the expression of A20, a tumor suppressor, which inhibits IKK's activity, therefore forming a delayed negative feedback on NFκB activity.

Hoffmann *et al*.'s work [19] showed that NFκB's expression level is oscillating in the nucleus after the cells are stimulated with TNFα. Our previous work applied parametric statistical model checking and simulation method [10] to verify NFκB's oscillation in a single cell in response to HMGB1 stimulus. Using the synchronous and asynchronous model checking methods, we can test this type of dynamic behaviors in the ER-Golgi network through verifying the following CTL formulas.

**Property 15: **PERK(IRE1) ≥ 1 → **AG**{(NFκB ≥ 1 → **AF**(NFκB = 0)) & (NFκB = 0 → **AF**(NFκB ≥ 1))}

**Property 16: **PERK(IRE1) ≥ 1 → **AG**{(NFκB ≥ 1 → **AF**(A20 ≥ 1)) & (A20 ≥ 1 → **AF**(NFκB = 0))}

**Property 17: AG**{(NFκB ≥ 1 → **AF**(NFκB = 0)) & (NFκB = 0 → **AF**(NFκB ≥ 1))}

**Property 18: AG**{(NFκB ≥ 1 → **AF**(A20 ≥ 1)) & (A20 ≥ 1 → **AF**(NFκB = 0))}

These formulas were verified to be true by the synchronous SMV checker. The properties 15-16 confirmed that, external stimulus, for example, overexpressed PERK or IRE1 under ER stress, could induce the oscillation of NFκB's expression in the nucleus. Properties 17-18 demonstrate that, nucleus NFκB oscillation does exist even if there is no external stimulus due to a self-contained NFκB-A20 negative feedback loop. However, these properties were falsified by the asynchronous model checker which searches and verifies all the possible reaction sequences that are non-deterministically chosen and executed. If the operator "**AF**" in the above formulas is replaced with "**EF**" (weaker than AF), the following oscillation properties 15'-18' were verified to be true by the asynchronous SMV (of course, they are also true in the synchronous one).

**Property 15': **PERK(IRE1) ≥ 1 → **AG**{(NFκB ≥ 1 → **EF**(NFκB = 0)) & (NFκB = 0 → **EF**(NFκB ≥ 1))}

**Property 16': **PERK(IRE1) ≥ 1 → **AG**{(NFκB ≥ 1 → **EF**(A20 ≥ 1)) & (A20 ≥ 1 → **EF**(NFκB = 0))}

**Property 17': AG**{(NFκB ≥ 1 → **EF**(NFκB = 0)) & (NFκB = 0 → **EF**(NFκB ≥ 1))}

**Property 18': AG**{(NFκB ≥ 1 → **EF**(A20 ≥ 1)) & (A20 ≥ 1 → **EF**(NFκB = 0))}

Finally, in the property 19 and 19', we predict a new phenomenon which describes another negative feed loop related to ATF4 in the cell cycle, and this property has not been observed in the wet lab experiments. Both formulas were verified by the synchronous SMV, but, only the formula 19' is verified to be true by the asynchronous checker.

**Property 19: AG**{(GADD34 = 2 → **AF**(ATF4 = 0)) & (ATF4 = 0 → **AF**(GADD34 = 0))}

**Property 19': AG**{(GADD34 = 2 → **EF**(ATF4 = 0)) & (ATF4 = 0 → **EF**(GADD34 = 0))}

## Conclusions

Experimental and clinical studies found that ER stress and malfunction of Golgi apparatus can activate both apoptosis and survival signaling pathways, which are implicated in the pathogenesis of Alzheimer's disease and cancer. Experimental biologists have recognized several proteins, which control the ER and Golgi homeostasis, as possible therapeutic targets for different diseases. The mechanisms that link ER stress and Golgi apparatus with the pathological changes of AD and cancer cells are still not very clear. Moreover, all the existing studies are based on the wet lab experiment or clinical observations. This work attempts to develop a computational model, which incorporates some signaling pathways that are activated due to ER stress, to study the roles of ER- Golgi network in the cell cycle progression. Due to the network complexity and many unknown parameters in the ER-Golgi network, we proposed and applied both synchronous and asynchronous symbolic model verification techniques to formally investigate these signaling pathways through verifying some temporal logic formulas, which abstractly encode the behaviors of some regulatory components in the cell of cancer and Alzheimer's disease.

Another novelty of this work is, the asynchronous symbolic model checking is, for the first time, proposed to qualitatively analyze the complex biological system and compare with existing experiments. Our previous studies [11,12,14] focused on the synchronous models which are not always correct in the cellular system. We remark that, since the asynchronous model checker executes the update of reactions asynchronously, that is, the choice of execution is nondeterministic, so some temporal logic properties verified by the synchronous SMV might not be true in the asynchronous model checking. Verification of temporal logic formulas in Alzheimer's disease indicates that, overexpressed/mutated TRAF2, NFκB or ASK1 in the cell will promote the synthesis of Amyloid-β (Aβ), leading to the pathogenesis of AD in the future. Asynchronous model checking also confirmed that continuous activation of TRAF2-JNK pathway might be associated with the Aβ synthesis. This result is consistent with the previous study in Alzheimer's disease [27], which found the level of TRAF2 was significantly higher in the frontal cortex of AD than the normal brains.

The properties related to cancer verified that, if S1P/S2P in the Golgi apparatus is continuously activated, it will continuously process ATF6 and activate the synthesis of the oncoproteins XBP1 and cMYC. If the cell is in the precancerous stage or some proteins are mutated, it can induce cancer cell's survival and inhibit apoptosis. Our work also identified several cancer-related genetic signatures in the ER-Golgi network, including IKK, NFκB and ATF4. Inflammation, which is partially regulated by the NFκB pathway, is associated with many chronic diseases, including cancer and Alzheimer's disease. Our work verified that, the nucleus NFκB's expression level is oscillating due to a negative feedback loop which is composed of oncoproteins (IKK, NFκB) and tumor suppressor (A20). This property has been verified by Hoffmann *et al*'s work [19]. Some predicted properties in this work could be tested in the future wet lab experiments.

Our technique has advantages for large network analysis and verification of both synchronous and asynchronous models over traditional simulation methods (it can check up to $10^{100}$ possible states). The computational model in this work is composed of ER stress-induced signaling pathways only, however, ER-Golgi network is a hub for both signaling pathways and also secretory pathways, which regulate the newly synthesized proteins' sorting and transportation. In our future studies, we will explore a larger model which incorporates both signaling pathways and secretory pathways to study the roles of ER and Golgi apparatus in the cell cycle, which might provide a new avenue to treat cancer and Alzheimer's disease through targeting the ER-Golgi-regulated pathways.

## Competing interests

The author declares that there are no competing interests.

## Authors' contributions

H.G. proposed the project, wrote the code and manuscript, H.G. and L.F. performed the verification.

## Declarations

Publication of this article was funded by the Saint Louis University.

This article has been published as part of *BMC Systems Biology *Volume 8 Supplement 4, 2014: Thirteenth International Conference on Bioinformatics (InCoB2014): Systems Biology. The full contents of the supplement are available online at http://www.biomedcentral.com/bmcsystbiol/supplements/8/S4.
